# A Study on the Stability and Carbohydrate Metabolic Traits of Starter Cultures in Response to Continuous Subculturing

**DOI:** 10.3390/ijms27062906

**Published:** 2026-03-23

**Authors:** Yangyang Yu, Jianjun Yang, Ran Wang, Lele Zhang, Kai Zhou, Baolei Li, Baochao Hou, Yue Sang, Haihong Feng, Yan Zhang, Jian He, Xiaoxia Li

**Affiliations:** 1 School of Food and Biological Engineering, Hefei University of Technology, Hefei 230601, China; 17855225489@163.com (Y.Y.); kaizhou@hfut.edu.cn (K.Z.); 2 Department of Nutrition and Health, Key Laboratory of Functional Dairy, Co-Constructed by Ministry of Education and Beijing Government, China Agricultural University, Beijing 100190, China; 18811626398@163.com (J.Y.); wangran@cau.edu.cn (R.W.); sy20243313942@cau.edu.cn (L.Z.); zhangyan@gsau.edu.cn (Y.Z.); 3 National Center of Technology Innovation for Dairy, Hohhot 100118, China; libaolei@yili.com (B.L.); hbch-1@163.com (B.H.); 4 Hebei Engineering Research Center of Animal Product, Sanhe 065200, China; sangyue2013@126.com (Y.S.); godlovefenghaihong@163.com (H.F.); 5 College of Food Science and Engineering, Gansu Agricultural University, Lanzhou 730070, China

**Keywords:** *S. thermophilus*, *L. bulgaricus*, continuous subculturing, physiological stability, genetic stability, carbon metabolism

## Abstract

The industrial application of starter cultures requires stable physiological and genetic performance. In this study, *Streptococcus salivarius* subsp. *thermophilus* and *Lactobacillus delbrueckii* subsp. *bulgaricus* were continuously subcultured. Physiological stability was assessed through colony morphology, fermentation activity, and growth profiling. Genetic stability was evaluated through comparative genomics of carbohydrate metabolism networks and single-nucleotide polymorphism (SNP) analysis. The results showed that after 2000 generations, the cellular morphology of the strains remained intact. Additionally, the strains exhibited enhanced growth performance and fermentation capability. The Gompertz model revealed that adapted *S. thermophilus* A37 and *L. bulgaricus* B29 exhibited shortened lag phases, increased maximum specific growth rates, and high stationary-phase cell densities. Phenotypic microarray and comparative genomics revealed that *S. thermophilus* mainly used mono- and disaccharides, with impaired ribose metabolism due to the absence of the *rbsk* gene in the pentose phosphate pathway. In contrast, *L. bulgaricus* metabolized diverse oligosaccharides, sugar alcohols, and plant-derived substrates. Additionally, it effectively catabolized ribose through the phosphoketolase pathway and possessed a trehalose degradation cluster. All strains exhibited genomic stability, with SNPs revealing fewer than 21 variations per isolate. This study provides an important theoretical foundation for evaluating the stability of fermentation starter cultures.

## 1. Introduction

Yogurt, a traditional fermented dairy product with a long history of consumption, has become a staple in the human diet owing to its distinctive flavor, smooth texture, and health benefits [1,2]. Yogurt is traditionally produced through the symbiotic fermentation of milk by *Streptococcus salivarius* subsp. *thermophilus* (*S. thermophilus*) and *Lactobacillus delbrueckii* subsp. *bulgaricus* (*L. bulgaricus*) [3]. In industrial applications, starter cultures are routinely subjected to prolonged preservation and repeated subculturing. During these processes, strains may accumulate genetic variations, undergo metabolic remodeling, and exhibit phenotypic changes, all of which can influence their fermentation performance. Furthermore, during production, strains encounter various environmental stresses, such as temperature fluctuations, osmotic shock, and pH variations [4]. These stresses act as selective pressures that can enrich for pre-existing genomic variations and induce adaptive regulatory responses, leading to altered metabolic regulation and phenotypic outcomes in lactic acid bacteria, thereby affecting their fermentation performance [5,6,7]. Therefore, developing an effective and stable evaluation system is crucial for the selection and application of fermentation starter strains.

Current methods for evaluating the stability of lactic acid bacteria strains are as follows: (1) cultivation under variable environmental conditions (e.g., temperature, pH, or nutrient composition) [8,9]; (2) exposure to extreme stress conditions, such as acidic, thermal, oxidative, or dehydrated environments [10]; and (3) continuous subculturing. Among these methods, continuous subculturing is widely used for assessing the physiological and genetic stability of lactic acid bacteria [11]. During long-term serial passage, strains accumulate genomic mutations and undergo transcriptional regulatory remodeling, thereby driving adaptive changes in metabolic pathways, carbon utilization efficiency, and environmental fitness [12]. Compared with short-term stress treatments, continuous subculturing under relatively stable growth conditions minimizes the interference of the confounding effects of transient environmental fluctuations [13]. This strategy facilitates a systematic evaluation of genetic architecture and physiological traits over long-term continuous subculturing [14]. However, most existing studies on the stability of lactic acid bacteria (LAB) have mainly focused on short-term stress responses, while the metabolic dynamics and genetic evolution of LAB under long-term serial passage remain unclear.

In this study, we systematically assessed the physiological and genetic stability of starter cultures under long-term serial passage conditions. Five *S. thermophilus* strains and three *L. bulgaricus* strains were continuously subcultured for 2000 generations. Morphological changes were first examined using scanning electron microscopy (SEM). In parallel, fermentation activity, growth kinetics, and metabolic capacity were assessed to comprehensively investigate the effects of prolonged cultivation on strain physiology. Subsequently, whole-genome sequencing was performed, followed by comparative genomic analyses to analyze characteristic carbon source metabolic networks and SNP variations, thereby revealing potential genetic changes. Overall, this study aimed to elucidate the stability patterns of *S. thermophilus* and *L. bulgaricus* under long-term passage conditions, providing theoretical insights to support their industrial application as fermentation starter cultures.

## 2. Results and Discussion

### 2.1. Morphological Changes in Fermentation Strains During Continuous Subculture

**Figure 1:**
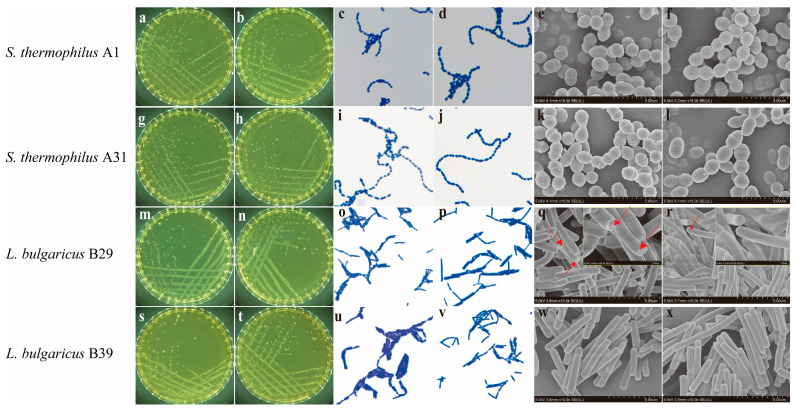
Colony and cellular morphology of *S. thermophilus* and *L. bulgaricus*: (**a**) colony morphology of wild-type *S. thermophilus* A1; (**b**) colony morphology after 2000 generations of continuous subculturing; (**c**) light microscopy image of wild-type *S. thermophilus* A1; (**d**) light microscopy after 2000 generations; (**e**) SEM image of wild-type *S. thermophilus* A1; (**f**) SEM image after 2000 generations. (**g**–**l**) *S. thermophilus* A4; (**m**–**r**) *L. bulgaricus* B29; (**s**–**x**) *L. bulgaricus* B39. For each strain group, the panel sequence is identical: colony morphology, light microscopy, and SEM image of the wild-type strain, followed by the corresponding image of the strain after 2000 generations of continuous subculture. Colony morphology images were photographed at a 1:1 scale without magnification. Light microscopy images were obtained using a 10× eyepiece and a 100× oil-immersion objective. SEM images were captured at 15,000× magnification for *S. thermophilus* and 10,000× magnification for *L. bulgaricus*. The red arrows in (**q**) indicate cell wall defects and fracture zones observable in the wild-type strain of *L. bulgaricus* B29. These defects have recovered after 2000 generations, as shown in (**r**).

The colony and cell morphology of *S. thermophilus* and *L. bulgaricus* were monitored throughout serial subculturing. Colonies of wild-type *S. thermophilus* displayed a milky-white to grayish-white appearance, with diameters ranging from 0.5 to 2 mm. They appeared round, convex, and well-defined and exhibited smooth and moist surfaces (Figure 1a,g and Figure S1). The diameter of colonies of wild-type *L. bulgaricus* was 1–3 mm, appearing as round, convex, milky-white colonies with smooth and moist surfaces (Figure 1m,s and Figure S1). Regarding cellular morphology, *S. thermophilus* mainly formed short- to medium-length chains (Figure 1c,i and Figure S1), whereas *L. bulgaricus* predominantly occurred in pairs, clusters, or short chains (Figure 1o,u and Figure S1). After 2000 generations of continuous subculturing, both the colony and cellular morphology of the strains remained unchanged. *S. thermophilus* colonies retained their milky-white to grayish-white color, diameter (0.5–2 mm), and smooth, convex, well-defined structure (Figure 1b,h and Figure S1), while the cells continued to form short- to medium-length chains (Figure 1d,j and Figure S1), showing no significant differences compared with the wild-type strains. Similarly, *L. bulgaricus* colonies remained round, convex, milky-white, and 1–3 mm in diameter, with smooth and moist surfaces (Figure 1n,t and Figure S1). Cells still occurred predominantly in pairs, clusters, or short chains (Figure 1p,v and Figure S1), consistent with the wild-type strains.

Morphological alterations during prolonged serial subculturing were further examined using SEM. After 2000 generations, the wild-type *S. thermophilus* A1 shifted from short chains composed of 2–5 cellular units to medium-long chains comprising 10–15 units (Figure 1e,f). In contrast, *S. thermophilus* A4, A31, A37, and A72 remained stable, retaining their original chain-length architecture (Figure 1k,l and Figure S1). Previous reports have shown that chain length in streptococci may be regulated by extracellular polysaccharides or autolysins. For example, Shibata et al. [15] identified the *atlA* gene in *Streptococcus mutans*, which encoded the major autolysin AtlA. The disruption of AtlA significantly reduced autolytic activity and resulted in impaired daughter cell separation, thus elongating cellular chains. SEM results for *L. bulgaricus* revealed that cells generally displayed smooth, well-defined surfaces and occurred primarily as single cells or short chains. However, a morphological change was observed in the specific wild-type strain *L. bulgaricus* B29. Initially, this strain exhibited visible cell wall defects and fractures (Figure 1q). Remarkably, after 2000 generations of serial subculture, its cellular morphology appeared more intact, with previously fractured surfaces showing signs of restoration, resulting in smoother and more defined structures (Figure 1r). This phenomenon indicates that prolonged subculturing under stable conditions contributed to enhanced cell wall integrity in this particular strain. In contrast, no such morphological deterioration or recovery was observed in the wild-type strains *L. bulgaricus* B39 and B43; these strains retained intact cellular morphology throughout 2000 passages (Figure 1w,x and Figure S1). The distinct morphological trajectory observed in strain B29 may be attributed to adaptive modifications in cell wall constituents, such as peptidoglycan, teichoic acids, and surface-associated proteins, which are known to modulate structural integrity [16]. This adaptive response is consistent with findings in other lactic acid bacteria, such as *Lactiplantibacillus plantarum*, which can upregulate the biosynthesis of structural cell wall components to mitigate cellular damage under stress [17].

### 2.2. Assessment of Growth Characteristics and Fermentation Activity After Continuous Subculture

After *S. thermophilus* and *L. bulgaricus* were continuously subcultured, the growth characteristics and fermentation performance of the strains were evaluated by measuring the pH of the culture medium and OD600 values. In wild-type *S. thermophilus* strains (A1, A4, A31, A37, and A72), the pH values ranged from 4.71 to 4.81, with OD600 values between 1.39 and 1.50 (Table 1). After 2000 generations of subculturing, the pH value ranged from 4.64 to 4.74, and OD600 values were between 1.39 and 1.55 (Table 1). Notably, the pH of *S. thermophilus* A37 significantly decreased from 4.81 to 4.64, while its OD600 significantly increased from 1.48 to 1.53 (*p* < 0.05) compared with the wild-type strain.

In contrast, the wild-type *L. bulgaricus* strains (B29, B39, and B43) showed lower medium pH values, ranging from 4.30 to 4.48, and higher OD600 readings between 1.99 and 2.10 (Table 1). After 2000 generations of subculturing, these strains demonstrated a further decrease in medium pH (4.07–4.33) and an increase in OD600 (2.06–2.19), indicating enhanced growth and acidification capacity relative to the *S. thermophilus* group. Among them, *L. bulgaricus* B29 exhibited a pH decrease of 0.23 and an increase in OD600 (0.09). Although the absolute pH difference may appear numerically small, a shift of 0.23 units corresponds to a substantial change in proton concentration, reflecting a physiologically meaningful enhancement in acid production. Collectively, the results indicate that long-term subculturing improved both acidification capacity and biomass accumulation in the evaluated strains.

Fermentation activity significantly increased in both species after 2000 generations (Table 1) (*p* < 0.05). Notably, after 2000 passages, the fermentation activity of *S. thermophilus* (A4 and A36) exceeded 60 U, while that of *L. bulgaricus* B29 and B43 was approximately 62.41 U and 61.9 U, respectively, indicating excellent fermentation performance. Prolonged adaptation of wild-type starter cultures in lactose-containing media can change key enzyme activities related to metabolic regulation and energy utilization efficiency, thereby enhancing environmental adaptability. For example, Yu et al. reported that elevated β-galactosidase and urease activities enhanced lactose metabolism in different *S. thermophilus* strains, improving fermentation performance [18].

### 2.3. Carbohydrate Metabolic Profiling

**Figure 2:**
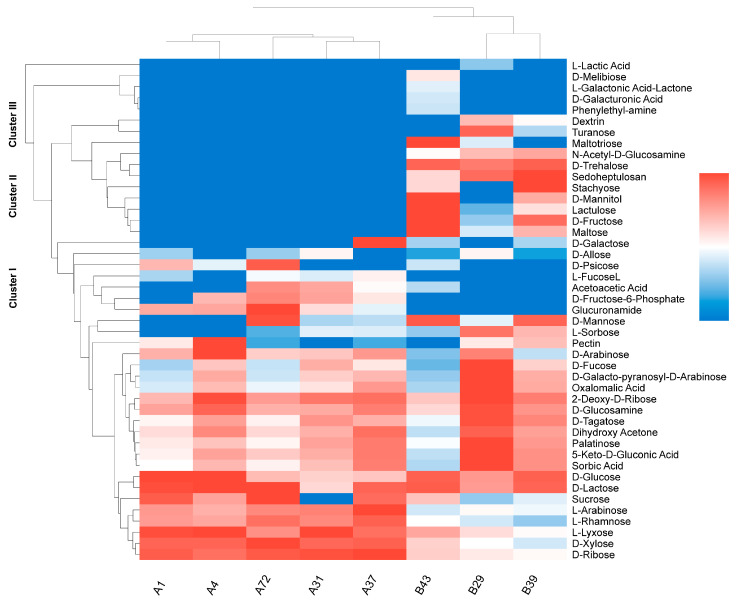
Heatmap and hierarchical cluster analysis of carbon source utilization by *S. thermophilus* and *L. bulgaricus* strains. The heatmap displays the phenotypic profiles of 46 carbon sources obtained using phenotype microarray (PM) technology. Color intensity represents relative utilization levels: red, high; white, moderate; blue, low or no utilization. Hierarchical clustering was performed using the UPGMA algorithm with Euclidean distance matrices, revealing three distinct metabolic clusters (Clusters I–III) that illustrate species- and strain-specific differences in carbohydrate metabolism between the two LAB.

Phenotypic MicroArray (PM) technology, a high-throughput platform that enables systematic assessment of cellular phenotypes such as carbon source utilization and metabolic activity, was used on the OmniLog™ system to comparatively analyze the carbon utilization profiles of *L. bulgaricus* across 46 distinct carbon sources. By analyzing the carbon utilization profiles of 2000 generations of strains, it was found that *S. thermophilus* utilized 28 carbon sources, while *L. bulgaricus* metabolized 43 carbon sources (Figure 2). The utilization profiles were clearly segregated into three distinct metabolic clusters (Clusters I–III).

Cluster I, co-driven by *S. thermophilus* and *L. bulgaricus*, consisted of monosaccharides, disaccharides, and metabolic intermediates. Monosaccharides in Cluster I included fundamental hexoses, such as D-glucose, D-galactose, and D-mannose, and pentoses, such as D-xylose, D-ribose, L-arabinose, and L-lyxose (Figure 2). In addition, Cluster I contained monosaccharide epimers, including epimers of glucose or fructose (e.g., D-allose, D-psicose, and D-tagatose) and 6-deoxy rare hexoses (e.g., L-fucose, D-fucose, and L-rhamnose) (Figure 2). Notably, *S. thermophilus* effectively metabolized L-arabinose, L-rhamnose, L-lyxose, and D-xylose, whereas *L. bulgaricus* weakly utilized these sugars (Figure 2). This difference might be attributed to the distinct ecological adaptations and genomic metabolic potentials of the two species. Cluster I contained some metabolic intermediates, such as D-fructose-6-phosphate and dihydroxyacetone. Dihydroxyacetone can be phosphorylated by dihydroxyacetone kinase and subsequently enter the EMP pathway for further metabolism [19].

Cluster II is mainly driven by *L. bulgaricus* and comprises oligosaccharides (e.g., dextrin, stachyose, maltotriose), functional disaccharides (e.g., maltose, turanose, lactulose), and sugar alcohols (Figure 2). The carbon sources in Cluster II are predominantly derived from plant or starch sources, and their utilization typically depends on starch hydrolysis systems involving key enzymes, including α-glucosidase [20,21] and trehalose-6-phosphate hydrolase [22]. Notably, D-trehalose serves as a carbon source and significantly enhances bacterial tolerance to processing stress, dehydration, and freezing conditions [23,24]. The carbon source utilization profile of Cluster II highlights the significant advantage of *L. bulgaricus* strains in metabolizing sugar alcohols and oligosaccharides, reflecting their adaptive capacity to complex environmental substrates.

Cluster III, distinct from clusters I and II, represents strains with specialized substrate-utilization capabilities, such as *L. bulgaricus* B43. This strain can utilize D-melibiose, L-galactonic acid-δ-lactone, D-galacturonic acid, and phenylethylamine (Figure 2). L-galactonic acid-δ-lactone is a characteristic intermediate produced during pectin decomposition, while D-galacturonic acid is a primary component of plant cell wall pectin. In prokaryotes, D-galacturonic acid is catabolized through two principal pathways: an oxidative route and an isomerization pathway [25,26]. Additionally, we observed that the carbohydrate utilization profiles of *S. thermophilus* and *L. bulgaricus* remained stable between wild-type strains and 2000 generations, indicating that their carbohydrate metabolic capabilities remained consistent.

### 2.4. Analysis of Microbial Growth Kinetics

**Figure 3:**
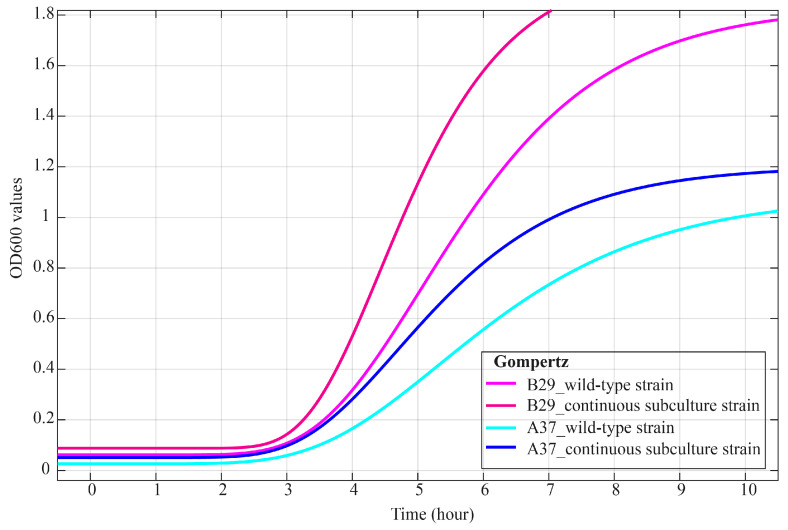
Gompertz model fitting of *S. thermophilus* A37 and *L. bulgaricus* B29 in wild-type strains and after 2000 generations of continuous subculturing. Growth curves were modeled using OD600 values.

Based on morphological observations, growth performance, fermentation activity, and carbohydrate metabolism profiling, *S. thermophilus* A37 and *L. bulgaricus* B29 were selected for microbial growth kinetics analysis. Both strains exhibited adaptive changes after long-term serial passage. The OD600 values of both wild-type (generation 0) and 2000-generation passaged strains were monitored at 1 h intervals to assess their growth curves. The Gompertz model was used to fit the growth curves of wild-type and 2000-generation passaged strains of *S. thermophilus* A37 and *L. bulgaricus* B29. The adjusted coefficient of determination (adjusted *R*^2^) and the root mean square error (RMSE) were used to assess the model performance. As shown in Table 2, all strains exhibited a high goodness of fit, with adjusted *R*^2^ values exceeding 0.995 (ranging from 0.996 to 0.997) and consistently low RMSE values ranging from 0.025 to 0.047. These results show that the Gompertz model accurately captures the growth dynamics of both species under the tested conditions and can be reliably used to compare kinetic parameters between wild-type and long-term subcultured strains. Growth curves of *S. thermophilus* A37 and *L. bulgaricus* B29, both in their wild-type and after 2000 generations of continuous subculturing, were obtained during monoculture in MRS medium with lactose substituted for glucose. The growth curves of both strains exhibited a characteristic asymmetric sigmoidal shape, with clearly distinguishable lag, exponential, and stationary phases (Figure 3 and Figure S1). For *S. thermophilus* A37, the lag phase duration (λ) decreased from 5.323 h in the wild-type strain to 4.688 h in the passaged strain. The corresponding viable counts at 3 h were 7.32 log_10_ CFU/mL in the passaged strain, compared with 7.20 log_10_ CFU/mL in the wild-type strain (Table 2). After entering the exponential phase, the maximum specific growth rate (*R*_m_) of the subcultured strain increased from 0.536 to 0.691, indicating accelerated growth (Table 2). Viable counts of the passaged strain were 8.38 log_10_ CFU/mL, significantly higher than that of the wild-type strain (7.90 log_10_ CFU/mL; *p* < 0.05) (Table 2). In the stationary phase, the maximum cell density (*N*_max_) was 1.203, with peak viable counts observed at 8 h (8.70 log_10_ CFU/mL for the passaged strain vs. 8.50 log_10_ CFU/mL for the wild-type).

The growth of *L. bulgaricus* B29 was more pronounced after long-term subculture. Compared with the wild-type strain, the lag phase duration (λ) of the 2000 generation strain reduced from 5.043 h to 4.427 h, and the *R*_m_ during the exponential phase increased from 0.634 to 0.887, representing a 28.8% increase. This phenomenon was further confirmed by microbial population dynamics. Although initial inoculum levels were similar, at the end of the lag phase (3 h), the viable counts of the passaged strain (6.78 log_10_ CFU/mL) exceeded those of the wild-type strain (6.24 log_10_ CFU/mL). During the exponential phase (approximately 5 h), viable counts of the passaged strain were 7.64 log_10_ CFU/mL, which were significantly higher than the 7.15 log_10_ CFU/mL recorded for the wild-type strain (*p* < 0.05). After the stationary phase (8 h), the viable counts of the passaged strain became 7.89 log_10_ CFU/mL. These results show that long-term continuous subculturing significantly enhances the growth performance of both *S. thermophilus* A37 and *L. bulgaricus* B29.

### 2.5. S. thermophilus and L. bulgaricus Genome Sequences

**Figure 4:**
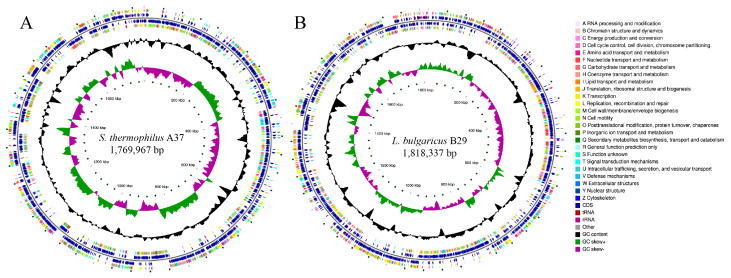
The circular map of *S. thermophilus* A37 and *L. bulgaricus* B29. (**A**) circular map of *S. thermophilus* A37; (**B**) circular map of *L. bulgaricus* B29. This circular genome visualization comprises six concentric rings. The first and fourth rings depict coding sequences (CDS) on the positive and negative strands, colored by COG category. The second and third rings show the positions of CDS, tRNA, and rRNA. The fifth ring displays GC content, with red peaks (outward) indicating values above the genome average and blue peaks (inward) below it; peak height reflects the magnitude of deviation. The sixth ring represents GC skew, which helps distinguish the leading from the lagging strand and locate the replication origin in circular genomes. The innermost ring indicates the genome size.

Genome sequencing of *S. thermophilus* and *L. bulgaricus* strains after 2000 generations of continuous subculturing revealed that the genome sizes of *S. thermophilus* isolates ranged from 1,769,967 to 1,789,462 bp, with GC contents between 38.93% and 38.95% (Figure 4A). In comparison, the genomes of *L. bulgaricus* strains ranged from 1,776,843 to 1,818,337 bp, with GC contents ranging from 49.81% to 49.85% (Figure 4B). These genomic characteristics are consistent with those reported for other commonly used starter strains. The genomic analysis revealed that *S. thermophilus* strains contained between 1882 and 1900 predicted coding sequences (CDSs) and 31–43 structural RNAs, including rRNA and tRNA (Table 3). Compared with *S. thermophilus*, *L. bulgaricus* strains possessed 1843–1877 CDSs and 81–90 structural RNAs (Table 3). No active prophages were detected within the genomic sequences of either *S. thermophilus* or *L. bulgaricus* using PHAST. GC content variation is widely believed to be a mutational bias. The genomic GC content of *S. thermophilus* was significantly lower than that of *L. bulgaricus*. The genome sequencing results of *S. thermophilus* were consistent with previous studies. For example, Kapse et al. reported that *S. thermophilus* MCC0200 possesses a circular chromosome of 1,855,815 bp with an average GC content of 39.1% [27]. Similarly, Alexandraki et al. analyzed 23 fully sequenced *S. thermophilus* genomes, which ranged from 1.73 to 2.10 Mb with an average GC content of 39.0% [28]. In contrast, *L. bulgaricus* strains generally have larger and more GC-rich genomes. Shehata et al. reported genome sizes of 1,752,493 bp and 1,759,908 bp with GC contents of 49.80% and 49.90% for strains *L. bulgaricus* CBC-LB69 and *L. bulgaricus* CBC-LB8 [29], respectively. Hao et al. found that *L. bulgaricus* 2038 has a genome length of approximately 1,872,907 bp and an average GC content of 49.68% [30]. Overall, the genomes of *L. bulgaricus* strains are larger and have higher GC content than those of *S. thermophilus*.

### 2.6. Differences in Carbon Source Metabolism Between S. thermophilus and L. bulgaricus

**Figure 5:**
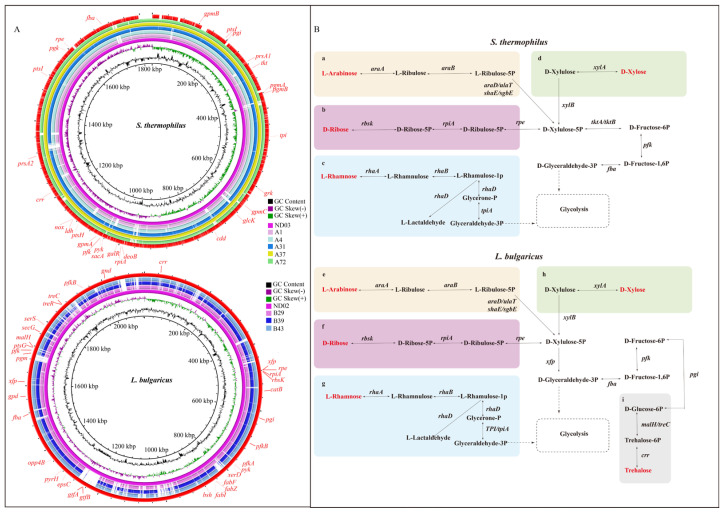
(**A**) Comparative analysis of carbohydrate metabolism–related genes in *S. thermophilus* and *L. bulgaricus*. The upper panel illustrates whole-genome comparisons of five *S. thermophilus* strains against the reference genome *S. thermophilus* ND03, while the lower panel compares three *L. bulgaricus* strains with the reference genome *L. bulgaricus* ND02. (**B**) Schematic representation of carbohydrate catabolic pathways in *S. thermophilus* and *L. bulgaricus*. Panels (**a**–**d**) correspond to pathways in *S. thermophilus*, whereas panels (**e**–**i**) correspond to those in *L. bulgaricus*. Yellow (L-arabinose), pink (D-ribose), blue (L-rhamnose), green (D-xylose), and gray (trehalose) modules represent the respective metabolic pathways. Solid arrows show metabolic flux direction, and dashed boxes highlight connections to glycolysis (EMP) and the PPP. The genes labeled (e.g., *araA*, *rhaD*, and *xylB*) denote key enzymes catalyzing the corresponding reactions.

After continuous subculturing of *S. thermophilus* and *L. bulgaricus* strains, a comparative genomic analysis was conducted on five *S. thermophilus* strains (A1, A4, A31, A37, and A72) and three *L. bulgaricus* strains (B29, B39, and B43), using *S. thermophilus* ND03 and *L. bulgaricus* ND02 as reference genomes. The analysis mainly focused on the utilization of carbon sources, with emphasis on the metabolism of D-ribose, L-arabinose, L-rhamnose, D-xylose, and trehalose. *S. thermophilus* generally retains the genes associated with pentose metabolism and central carbon metabolism, including the pentose phosphate pathway (PPP), such as *tktA/B* (transketolase), *pfk* (phosphofructokinase), and *fba* (aldolase), which encode the corresponding enzymes and collectively constitute the core metabolic module of the PPP (Figure 5A). The catabolic pathways of D-ribose, L-arabinose, and D-xylose converged into the PPP through the intermediate xylulose-5-phosphate (Xu5P). Xu5P was subsequently funneled into central carbon metabolism, where transketolase- and transaldolase-catalyzed reactions converted PPP intermediates into D-fructose-6-phosphate (F6P) through the catalysis of transketolase (*tktA/B)*. F6P was phosphorylated by phosphofructokinase (*pfk*) to generate D-fructose-1,6-bisphosphate (F1,6BP). Finally, aldolase (*fba*) catalyzed the cleavage of F1,6BP into glyceraldehyde-3-phosphate and dihydroxyacetone phosphate (Figure 5B). In *S. thermophilus*, the D-ribose metabolic pathway was facilitated by *rpiA* and *rpe* genes. These genes encoded enzymes that catalyzed the conversion of D-ribose-5-phosphate and D-ribulose-5-phosphate into D-xylulose-5-phosphate, which subsequently entered the PPP. However, the *rbsK* gene was absent in *S. thermophilus*, thereby limiting its ability to initiate complete ribose catabolism.

In contrast, *L. bulgaricus* retained a complete D-ribose catabolic gene cluster (*rbsK*, *rpiA*, and *rpe*) (Figure 5A). The resulting metabolic flux sequentially proceeded through phosphorylation, isomerization, and epimerization to generate Xu5P, which was then cleaved by xylulose-5-phosphate phosphoketolase (*xfp*) into G3P and acetyl-phosphate, both of which entered central pyruvate metabolism (Figure 5B). Additionally, *L. bulgaricus* retained multiple genes associated with the metabolism of plant-derived sugars, particularly those involved in trehalose utilization, including *crr*, *malH/treC*, *pgi*, *pfk*, and *fba* (Figure 5A). Trehalose was imported and phosphorylated by the trehalose-specific PTS permease (*crr*). The resulting trehalose-6-phosphate was hydrolyzed by trehalose-6-phosphate hydrolase (*malH*/*treC*) to glucose and G6P. Then, G6P was sequentially metabolized by the *pgi*, *pfk*, and *fba* gene products, leading to the generation of C3 intermediates that feed into the pyruvate pathway (Figure 5B). Comparative genomic analysis between *S. thermophilus* and *L. bulgaricus* revealed distinct pentose metabolism strategies. Although *S. thermophilus* mainly uses the classic transketolase-dependent PPP, *L. bulgaricus* uses a more efficient phosphoketolase bypass that directly channels pentoses into central metabolism with fewer enzymatic steps [31], reflecting its evolutionary adaptation to dairy environments. Additionally, *L. bulgaricus* harbors a trehalose degradation gene cluster, enabling it to utilize the stress-protective sugar to enhance tolerance under adverse conditions, such as acid, cold, or desiccation [32,33].

### 2.7. Genetic Stability After Continuous Subculture

Whole-genome resequencing (average depth ≈ 1000×) was performed on *S. thermophilus* strains (A1, A4, A31, A37, and A72) and *L. bulgaricus* strains (B29, B39, and B43) after 2000 generations of continuous subculturing to identify SNPs and other genetic variations. As shown in Table 4, genomic alterations, including point mutations, insertions, deletions, and multiple nucleotide polymorphisms (MNPs), were detected in all *S. thermophilus* strains. For example, *S. thermophilus* A37 exhibited six SNPs, three MNPs, three insertions, and two deletions, whereas *S. thermophilus* A72 exhibited fewer variations, comprising four SNPs and two deletions. Similarly, *L. bulgaricus* strains exhibited comparable genomic variations. Among the strains, *L. bulgaricus* B39 presented the highest number of variations, a total of 15 SNPs, three MNPs, and one deletion, while *L. bulgaricus* B43 contained 12 SNPs, two insertions, and four deletions. Overall, the total number of genetic variations in *S. thermophilus* (A1, A4, A31, A37, and A72) and *L. bulgaricus* (B29, B39, and B43) remained limited after 2000 generations of continuous subculturing. Pightling et al. [34] proposed that fewer than 21 SNP differences in whole-genome sequencing data could be used as a threshold to support the conclusion that two or more bacterial genomes were closely related or identical. Our results meet this criterion, indicating that the subcultured strains retain high genomic identity with their progenitors, thus exhibiting excellent genetic stability under the experimental conditions.

Functional annotation and pathway enrichment analyses indicated that the identified variations (totaling fewer than 20 SNPs across all strains) were distributed among genes involved in diverse cellular processes, including basic metabolism and stress response. Importantly, no mutations were found in key genes or known regulatory elements directly associated with the pentose phosphate pathway (PPP) or the phosphoketolase pathway (PKP). Therefore, the current mutational profile does not support the notion that genetic mutations directly drove adaptive changes in the activity or flux of these specific pathways during long-term subculturing.

## 3. Materials and Methods

### 3.1. Continuous Subculturing of S. thermophilus and L. bulgaricus

The strains were inoculated at 2% (*v*/*v*) into the MRS medium in which lactose replaced glucose at a pH of 6.5. Serial subculturing was performed, with transfers conducted during the exponential growth phase, until the cumulative number of cell divisions reached approximately 2000 generations. The generation time (*g*) of *Streptococcus salivarius* subsp. *thermophilus* (*S. thermophilus*) and *Lactobacillus delbrueckii* subsp. *bulgaricus* (*L. bulgaricus*) during the exponential growth phase was calculated according to the following formula:

(1) $$g\;=\;\frac{t\cdot \mathrm{lg}2}{\mathrm{lg}N_{t}-\mathrm{lg}N_{0}}$$

where *t* represents the cultivation time (h), *N*_0_ is the initial viable cell count (CFU/mL), *N_t_* is the viable cell count (CFU/mL) at time *t*, and *g* denotes the generation time (h).

### 3.2. Measurement of pH, TA, and Viable Counts

Optical density (OD_600_) of the bacterial cultures was measured at 600 nm every 400 generations using a UV-2800A spectrophotometer (Unico Instrument Co., Ltd., Beijing, China). The pH of the culture sample was directly measured with a calibrated pH meter (Model S210, Mettler Toledo, Zurich, Switzerland). Viable counts of *S. thermophilus* and *L. bulgaricus* were determined by plating appropriate dilutions on M17 agar and MRS agar at an adjusted pH of 5.4, respectively. Plates were incubated at 42 °C for 48 h, and results were expressed as colony-forming units per gram of sample (CFU/g) [35]. All analyses were performed in triplicate using independently cultured samples.

### 3.3. Fermentation Vitality

Fermentation activity of strains was assessed every 400 passages during continuous subculturing, with all assays performed in triplicate. Activity was assessed by measuring the amount of acid produced during carbohydrate fermentation. Bacterial cells were inoculated into 10 mL of sterile skim milk at a concentration of 1 × 10^7^ CFU/mL and incubated at 42 °C for 24 h. After the incubation was complete, the samples were immediately cooled in an ice bath to terminate fermentation and then diluted with 20 mL of distilled water. The pH of the diluted samples was adjusted to 8.50–8.55 with 0.1 mol/L NaOH, and the endpoint was held for 30 s. The volume of NaOH consumed was recorded to calculate the lactic acid concentration, which was converted into activity units. One activity unit (U) was defined as the amount of lactic acid (1 μmol) produced by 10^7^ cells during the fermentation of 1 mL of skim milk under the described conditions.

### 3.4. Scanning Electron Microscopy (SEM) Analysis

Subcultured strains were continuously harvested through centrifugation at 1000× *g* for 10 min. After the subcultured strains were washed twice with 10 mmol/L of phosphate-buffered saline (137 mM NaCl, 2.7 mM KCl, 10 mM Na_2_HPO_4_, and 2 mM KH_2_PO_4_; pH 7.4), the cell pellet was resuspended to a final concentration of 1 × 10^5^ CFU/mL. The cell suspension was fixed with 2.5% (*v*/*v*) glutaraldehyde and incubated at 4 °C for 24 h. After the mixture was centrifuged, the bacterial pellet was sequentially dehydrated with 30%, 50%, 70%, and 100% ethanol. The samples were lyophilized and subsequently gold-coated under vacuum (4.0 Pa) using an ion sputter coater (Hitachi MC1000, Hitachi High-Tech Corporation, Tokyo, Japan). A cold field-emission scanning electron microscope (Hitachi SU8010) operated at an accelerating voltage of 5 kV and a beam current of 10 μA was used to examine the morphology of the samples.

### 3.5. Growth Kinetics Modeling

The growth kinetics of *S. thermophilus* A37 and *L. bulgaricus* B29 were described using the Gompertz model. The four-parameter model is suitable for fitting asymmetric sigmoidal growth curves, characterized by initial lag, exponential growth, and stationary phases. The modified Gompertz equation was applied as follows:

(2) $$N\left(t\right)\;=\;N_{0}\;+\;N_{m}*\exp(-\exp(\frac{R_{m}*e}{N_{m}}\;+\;1))$$

where *N*(*t*) is the cell density (OD_600_) at time *t* (h); *N*_0_ is the initial cell density; *N*_m_ is the maximum cell density; λ is the lag phase duration (h); *R*_m_ is the maximum specific growth rate (h^−1^); and *e* is the base of the natural logarithm.

### 3.6. Whole-Genome Sequencing and Comparative Genome Analysis

Genomic DNA was extracted using the NEXTFLEX Rapid DNA-Seq Kit according to the manufacturer’s instructions. Drafts and complete genomes of five *S. thermophilus* and three *L. bulgaricus* strains were sequenced using the PacBio Sequel (Pacific Biosciences of California, Inc., Menlo Park, CA, USA)and the Illumina NovaSeq PE150 platform (Illumina, Inc., San Diego, CA, USA). Coding sequences (CDSs) were functionally annotated against the non-redundant, Swiss-Prot, Pfam, Gene Ontology, Clusters of Orthologous Genes (COG), and Kyoto Encyclopedia of Genes and Genomes (KEGG) databases with sequence alignment tools using BLASTP (NCBI BLAST+ version 2.17.0), Diamond, and HMMER. For each predicted protein, the annotation of the highest-scoring hit (e-value < 10^−5^) was retained. Genome-wide similarity was assessed using OrthoANIu (OrthoANI using USEARCH). Comparative genomic visualization was performed using the BLAST-based Circular Image Generator (v0.95).

### 3.7. Phenotypic Analyses

Carbon source utilization profiles of five *S. thermophilus* and three *L. bulgaricus* strains were analyzed using the Phenotype MicroArray system (Biolog, Hayward, CA, USA). PM1 and PM2A microplates were used to evaluate the metabolic capabilities of the strains toward different carbon sources [36]. Following the manufacturer’s protocol, the growth on MRS agar was suspended in IF-0a inoculating fluid to obtain a cell suspension with 81% light transmittance. Aliquots of 100 μL were dispensed into the wells of 96-well Phenotype MicroArray (PM) plates. Then, the plates were incubated in an OmniLog reader at 42 °C for 72 h. During incubation, the absorbance of the redox-sensitive dye G was automatically recorded every 15 min. Data were processed and analyzed using the OmniLog PM software 2.3.01 (Biolog) according to the manufacturer’s instructions.

### 3.8. Statistical Analysis and Genome Submission

Functional classification of protein-coding genes was performed using BLASTP (E-value ≤ 10^−5^) against NCBI COGs (Clusters of Orthologs), with a minimum identity of ≥30% and coverage of ≥30%. Sequence similarity was detected using BLAST, and multiple sequence alignments were conducted using Clustal 2.1. Putative carbohydrate metabolic pathways were predicted using KEGG. Data from growth characteristics, fermentation vitality, and phenotypic microarray analyses were analyzed using one-way analysis of variance (ANOVA). Each experiment was performed in triplicate, and pairwise comparisons of treatment means were conducted using Tukey’s test at *p* < 0.05 with Statistica, SPSS (27.0), and GraphPad Prism 8.0. All assembled genomes were submitted to the NCBI GenBank database (https://www.ncbi.nlm.nih.gov/) and assigned accession numbers. Strain designations and GenBank accession numbers were as follows: *S. thermophilus* A1 (PX945674); *S. thermophilus* A4 (PX945677); *S. thermophilus* A31 (PX945682); *S. thermophilus* A37 (PX945711); *S. thermophilus* A72 (PX945712); *L. bulgaricus* B29 (PX944889); *L. bulgaricus* B39 (PX944890); *L. bulgaricus* B43 (PX945891). Genome data of the reference genomes used for genome comparison were *S*. *thermophilus* ND03 (CP002340) and *L. bulgaricus* ND02 (CP002341.1).

## 4. Conclusions

In this study, after continuous subculturing for 2000 generations, both *S. thermophilus* and *L. bulgaricus* strains maintained stable colony and cellular morphology and retained high fermentative activity. Carbon utilization profiling revealed that *S. thermophilus* metabolized 28 carbon sources, including monosaccharides and disaccharides (e.g., L-arabinose), while *L. bulgaricus* utilized 43 substrates, including oligosaccharides, functional disaccharides, and sugar alcohols. Long-term subculturing significantly enhanced growth kinetics, which were confirmed by shortened lag phases, elevated maximum specific growth rates, and increased stationary-phase cell densities. Comparative genomic analysis revealed divergent metabolic strategies: *L. bulgaricus* used an efficient phosphoketolase-dependent bypass for pentose catabolism and possessed a trehalose degradation cluster, whereas *S. thermophilus* relied on the canonical transketolase-driven PPP. Whole-genome resequencing further confirmed that both species exhibited high genomic identity with their ancestral strains after prolonged subculturing, indicating robust genetic stability suitable for industrial applications. However, the mere presence of genes does not necessarily guarantee functional expression or metabolic flux. Further validation through transcriptomic, proteomic, enzymatic, or metabolic flux analyses would provide deeper insight into the active mechanisms underlying these metabolic pathways.

## Figures and Tables

**Table 1:** Growth characteristics of *S. thermophilus* and *L. bulgaricus* in the wild-type strain and generation 2000 during continuous subculturing. One viability unit (U) is defined as 1 μmol of lactic acid per 1 × 10^7^ CFU of bacterial cells during the fermentation of 1 mL of milk under the above conditions. All assays were performed in triplicate, and data are expressed as mean ± standard deviation (SD). Values in the same row that share a lowercase letter (a, b) are not significantly different; different letters indicate statistically significant differences (*p* < 0.05) between 0 generations and after 2000 generations of the same strain, as determined by one-way ANOVA followed by Tukey’s multiple comparison test.

Generation Time (n)	pH Values		OD600		Fermentation Activity (U)	
	0	2000	0	2000	0	2000
A1	4.73 ± 0.03 ^a^	4.69 ± 0.01 ^a^	1.37 ± 0.02 ^a^	1.49 ± 0.01 ^b^	45.33 ± 1.02 ^a^	51.37 ± 0.80 ^b^
A4	4.81 ± 0.01 ^a^	4.74 ± 0.01 ^a^	1.49 ± 0.01 ^a^	1.52 ± 0.01 ^b^	57.41 ± 0.98 ^a^	64.10 ± 2.06 ^b^
A31	4.75 ± 0.00 ^a^	4.66 ± 0.02 ^b^	1.39 ± 0.01 ^a^	1.39 ± 0.02 ^a^	50.27 ± 1.56 ^a^	56.29 ± 1.14 ^b^
A37	4.81 ± 0.00 ^a^	4.64 ± 0.01 ^b^	1.48 ± 0.02 ^a^	1.53 ± 0.01 ^b^	50.86 ± 0.91 ^a^	58.95 ± 1.64 ^b^
A72	4.71 ± 0.00 ^a^	4.66 ± 0.01 ^b^	1.50 ± 0.00 ^a^	1.55 ± 0.02 ^a^	52.10 ± 1.28 ^a^	55.65 ± 0.51 ^b^
B29	4.30 ± 0.00 ^a^	4.07 ± 0.01 ^b^	2.10 ± 0.01 ^a^	2.19 ± 0.01 ^b^	57.01 ± 0.42 ^a^	62.41 ± 0.67 ^b^
B39	4.32 ± 0.01 ^a^	4.31 ± 0.01 ^a^	2.00 ± 0.01 ^a^	2.06 ± 0.01 ^b^	33.10 ± 0.78 ^a^	41.69 ± 1.55 ^b^
B43	4.48 ± 0.01 ^a^	4.33 ± 0.01 ^b^	1.99 ± 0.01 ^a^	2.08 ± 0.01 ^b^	56.38 ± 2.01 ^a^	61.90 ± 1.72 ^b^

**Table 2:** Growth kinetic parameters of *S. thermophilus* A37 and *L. bulgaricus* B29 were determined in both wild-type strains and after 2000 generations of continuous subculturing. Growth dynamics were modeled using the Gompertz equation. The analyzed parameters included *N*_max_ (maximum cell density, corresponding to OD600), *R*_m_ (maximum specific growth rate), λ (lag phase duration, h), *N*_0_ (initial cell density), root mean square error (RMSE), and adjusted *R*^2^ (adjusted coefficient of determination). Bacterial counts were measured at different cultivation times (0, 2, 3, 5, and 8 h). All experiments measuring viable cell counts were performed in triplicate, and data are expressed as mean ± standard deviation (SD). Within the same row, different lowercase letters (a, b) indicate significant differences in viable cell counts at different time points for the same strain (*p* < 0.05).

Gompertz	A37-0	A37-2000	B29-0	B29-2000
*N*_max_	1.089	1.203	1.837	1.998
*R*_m_	0.536	0.691	0.634	0.887
λ (h)	5.323	4.688	5.043	4.427
*N*_0_	0.026	0.050	0.061	0.087
SSE	0.005	0.007	0.013	0.015
Adjusted *R*^2^	0.996	0.996	0.997	0.997
RMSE	0.025	0.031	0.043	0.047
0 h (log_10_ CFU/mL)	6.77 ± 0.01 ^a^	6.80 ± 0.02 ^a^	5.43 ± 0.01 ^a^	5.51 ± 0.02 ^a^
2 h (log_10_ CFU/mL)	6.71 ± 0.05 ^a^	6.89 ± 0.02 ^b^	5.75 ± 0.03 ^a^	6.15 ± 0.01 ^b^
3 h (log_10_ CFU/mL)	7.20 ± 0.05 ^a^	7.32 ± 0.02 ^b^	6.24 ± 0.04 ^a^	6.78 ± 0.02 ^b^
5 h (log_10_ CFU/mL)	7.90 ± 0.05 ^a^	8.38 ± 0.05 ^b^	7.15 ± 0.06 ^a^	7.64 ± 0.04 ^b^
8 h (log_10_ CFU/mL)	8.50 ± 0.02 ^a^	8.70 ± 0.07 ^b^	7.75 ± 0.05 ^a^	7.89 ± 0.03 ^b^

**Table 3:** The general genomic characteristics of five *S. thermophilus* and three *L. bulgaricus* strains. The table includes assembly statistics and functional annotations based on COG and KEGG subsystems. Functional comparison of metabolic genes across these strains was performed using Diamond, with an E-value cutoff set at ≤1 × 10^−5^.

	A1	A4	A31	A37	A72	B29	B39	B43
**General genome feature**								
Size	1,784,905	1,785,671	1,789,462	1,769,967	1,781,421	1,818,337	1,812,629	1,776,843
GC content	38.95	38.95	38.94	38.93	38.99	49.84	49.85	49.81
CDS No.	1889	1890	1890	1882	1900	1877	1869	1843
Number of RNAs	41	31	37	43	43	90	86	81
**Subsystem features**								
COG gene No.	1574	1572	1567	1552	1595	1486	1485	1489
Percent of All Genes (%)	83.32	83.17	82.91	82.47	83.95	79.17	79.45	80.79
**KEGG Pathway Enrichment**								
Carbohydrate metabolism	109	111	110	106	105	139	123	127
Amino acid metabolism	124	128	124	122	118	142	102	95
Metabolism of other amino acids	25	26	25	26	25	35	31	32
Energy metabolism	46	46	49	46	47	53	65	62
Lipid metabolism	32	33	32	31	30	37	44	44
Nucleotide metabolism	67	67	67	66	68	75	78	79

**Table 4:** Genomic mutation profiles of *S. thermophilus* and *L. bulgaricus* in the wild-type strain and generation 2000 during continuous subculturing.

Gene Mutation Type	Strains							
	A1	A4	A31	A37	A72	B29	B39	B43
Single-nucleotide polymorphism (SNP)	6	10	5	6	4	10	15	12
Multiple nucleotide polymorphism (MNP)	0	0	2	3	0	1	3	0
Insertion mutation (INS)	2	1	4	3	0	0	0	2
Deletion mutation (DEL)	0	1	0	2	2	1	1	4
Inversion mutation (INV)	0	0	0	0	0	0	0	0
Duplicate mutation (DUP)	0	0	0	0	0	0	0	0
Balanced chromosomal translocation (BED)	0	0	0	0	0	0	0	0
Intergenic genomic variation	0	0	0	0	0	0	0	0

## Data Availability

The original contributions presented in this study are included in the article/Supplementary Materials. Further inquiries can be directed to the corresponding authors.

## Supplementary Materials

The following supporting information can be downloaded at: https://www.mdpi.com/article/10.3390/ijms27062906/s1.
