# Supplementary material for: A Study on the Stability and Carbohydrate Metabolic Traits of Starter Cultures in Response to Continuous Subculturing
Source: Int J Mol Sci. 2026 Mar 23;27(6):2906. doi: 10.3390/ijms27062906 (PMC13027167; doi:10.3390/ijms27062906)
Supplement: Supplementary file 1 [file ijms-27-02906-s001.zip › Figure S1.pdf]

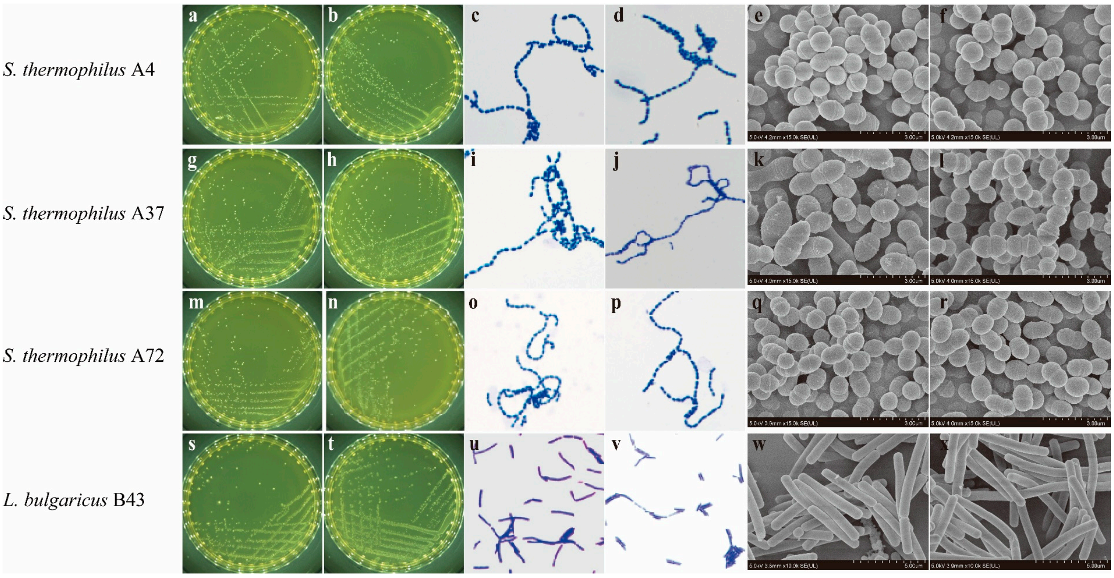

Figure S1. Colony and cellular morphology of *S. thermophilus* and *L. bulgaricus*. (a) Colony morphology of wild-type *S. thermophilus* A4; (b) colony morphology after 2000 generations of continuous subculture; (c) light microscopy image of wild-type *S. thermophilus* A4; (d) light microscopy after 2000 generations; (e) SEM image of wild-type *S. thermophilus* A4; (f) SEM image after 2000 generations. (g–l) *S. thermophilus* A37; (m–r) *S. thermophilus* A72; (s–x) *L. bulgaricus* B43. For each strain group, the panel sequence is identical: colony morphology, light microscopy, and SEM image of the wild-type strain, followed by the corresponding image of the strain after 2000 generations of continuous subculture. Colony morphology images were photographed at a 1:1 scale without magnification. Light microscopy images were obtained using a 10× eyepiece and a 100× oil-immersion objective. SEM images were captured at 15,000× magnification for *S. thermophilus* and 10,000× magnification for *L. bulgaricus*.
