# Supplementary material for: A Study on the Stability and Carbohydrate Metabolic Traits of Starter Cultures in Response to Continuous Subculturing
Source: Int J Mol Sci. 2026 Mar 23;27(6):2906. doi: 10.3390/ijms27062906 (PMC13027167; doi:10.3390/ijms27062906)
Supplement: Supplementary file 1 [file ijms-27-02906-s001.zip › Table S1.pdf]

Table S1. Changes in OD_{600} values of *S. thermophilus* A37 and *L. bulgaricus* B29 during cultivation. The wild-type strains (0 generation) and strains subjected to long-term continuous subculturing (2000 generations) were monitored. OD_{600} was measured at 1 h intervals during cultivation. Data are presented as the mean ± standard deviation of three biological replicates (n = 3).

Culture time (h)	*S. thermophilus* A37-0	*S. thermophilus* A37-2000	*L. bulgaricus* B29-0	*L. bulgaricus* B29-2000
0	0.026 ± 0.007	0.024 ± 0.003	0.046 ± 0.002	0.055 ± 0.004
1	0.022 ± 0.002	0.025 ± 0.005	0.045 ± 0.003	0.054 ± 0.003
2	0.033 ± 0.005	0.036 ± 0.005	0.063 ± 0.004	0.108 ± 0.002
3	0.066 ± 0.006	0.100 ± 0.009	0.137 ± 0.018	0.216 ± 0.034
4	0.151 ± 0.020	0.269 ± 0.030	0.342 ± 0.015	0.481 ± 0.001
5	0.338 ± 0.047	0.573 ± 0.043	0.675 ± 0.061	1.154 ± 0.002
6	0.591 ± 0.055	0.832 ± 0.041	1.031 ± 0.045	1.615 ± 0.001
7	0.741 ± 0.104	0.992 ± 0.069	1.467 ± 0.048	1.779 ± 0.001
8	0.815 ± 0.106	1.072 ± 0.074	1.600 ± 0.057	1.883 ± 0.007
9	0.958 ± 0.070	1.108 ± 0.046	1.690 ± 0.047	1.978 ± 0.004
10	1.023 ± 0.055	1.219 ± 0.011	1.734 ± 0.072	2.007 ± 0.001
